# microRNA‐7‐5p and α‐Synuclein SAA Predict Parkinson's Disease Phenoconversion

**DOI:** 10.1002/acn3.70539

**Published:** 2026-09-25

**Authors:** Shayan Zadegan, Praveen Velammal, Kanmani Muthiah, Madhu Jatty, Christopher Adams

**Affiliations:** ^1^ Department of Neurology The University of Tennessee Health Science Center Memphis Tennessee USA

**Keywords:** alpha‐synuclein seed amplification assay, microRNA‐7‐5p, neuron‐derived extracellular vesicle alpha‐synuclein, Parkinson's disease, prodromal Parkinson's disease

## Abstract

**Objective:**

Corroborate blood neuron‐derived extracellular vesicle (NDEV) alpha‐synuclein (αSyn), the CSF αSyn seed amplification assay (αSyn‐SAA), and blood microRNA‐7‐5p (miR‐7‐5p) as markers for Parkinson's disease (PD) phenoconversion and determine if combining these markers would help select subjects who would be more likely to phenoconvert.

**Methods:**

Using Parkinson Progression Marker Initiative data, time to PD phenoconversion was determined by Hoehn and Yahr scores. Log‐rank analysis was used to find cutpoints. Cox regression analysis was used with log[miR‐7‐5p], log[NDEV αSyn], and the αSyn‐SAA. Models included age and sex.

**Results:**

In a Cox model with baseline log[miR‐7‐5p], for each unit increase in log[miR‐7‐5p], there was 1.57 times increased PD phenoconversion (*p* = 0.003). In a model with baseline log[NDEV αSyn], only baseline age was associated with increased phenoconversion (*p* < 0.05). Using Cox regression analysis, the cutpoints for miR‐7‐5p and NDEV αSyn were 27.39 reads per million mapped (*p* < 0.01) and 18.36 pg/mL (*p* = 0.01), respectively. In separate models using log[miR‐7‐5p] and log[NDEV αSyn] cutpoints, higher levels of log[miR‐7‐5p] and log[NDEV αSyn] resulted in 2.26 (*p* < 0.001) and 4.46 (*p* < 0.05) times increased PD phenoconversion, respectively. A positive αSyn‐SAA was associated with 3.62 times increased phenoconversion (*p* < 0.001). Having log[miR‐7‐5p] higher than its cutpoint and a positive αSyn‐SAA was associated with 4.31 times phenoconversion (*p* < 0.001).

**Interpretation:**

Blood miR‐7‐5p, NDEV αSyn, and αSyn‐SAA could be used to enrich for prodromal patients likely to develop PD earlier in disease‐modifying clinical trials and combining them could further enrich for this population.

## 1 Introduction

Parkinson's disease (PD) is the second most common neurodegenerative disease and fastest growing neurological disease in prevalence and disability [1, 2]. The prevalence of PD is forecasted to be 267 cases per 100,000 by 2050, which would be a 76% increase from 2021 [3]. PD is a major source of financial burden on healthcare systems and psychological burden for caregivers [4, 5]. Pathologically, PD is characterized by aggregation of α‐synuclein (αSyn) in neurons, which in turn leads to neuronal degeneration in different regions of the brain. Non‐motor symptoms like REM behavior disorder (RBD), constipation, and anosmia occur prior to the appearance of motor symptoms during the prodromal phase of the disease. It is estimated that motor signs only appear when about 30%–50% of substantia nigra neurons are lost [6]. Hence it is important to identify clinical, imaging, and blood biomarkers that will help identify patients with prodromal PD and patients at risk of more rapid phenoconversion before significant neuronal loss occurs.

The CSF αSyn seed amplification assay (αSyn‐SAA) is a method of detecting whether pathological αSyn fibrils are present. It was found that this method could identify prodromal PD patients more likely to phenoconvert [7]. However, this method was not as accurate in identifying individuals with genetic mutations more likely to phenoconvert. Further, it requires a lumbar puncture. It would be preferable to have blood biomarkers for selecting individuals more likely to phenoconvert sooner.

Extracellular vesicles (EVs) are cell‐derived membranous particles that play an important role in molecular trafficking, intercellular transport, and elimination of unwanted proteins. EVs carry cell fingerprint proteins and microRNAs (miRNAs) and are diagnostically relevant to neurodegenerative disease [8, 9]. Serum neuron‐derived extracellular vesicles (NDEVs) are a potential alternative to CSF assays that would not require a lumbar puncture. NDEVs can be extracted from serum using the L1CAM antibody. Serum NDEV αSyn has been found to be upregulated in prodromal PD patients more likely to phenoconvert to PD [10].

Compared to NDEV αSyn or the αSyn‐SAA, microRNA‐7‐5p (miR‐7‐5p) could be more effective in identifying prodromal patients with genetic mutations, such as LRRK2. miR‐7‐5p has been found to regulate αSyn aggregation and translation [11, 12, 13] as well as NLRP3 inflammasome activation [14]. miR‐7‐5p has been found to be downregulated in the substantia nigra in people with Parkinson's disease (PwP) [15]. Further, it was found to decrease with progression in PwP compared to healthy controls (HCs) [16, 17]. On the other hand, miR‐7‐5p has been found to be upregulated in EVs from prodromal PwP who phenoconvert to PD more rapidly [18].

The purpose of this study is to corroborate previous studies looking at αSyn‐SAA, NDEV αSyn, and blood miR‐7‐5p as prodromal biomarkers and to look at whether the combination of these markers could improve selection of prodromal PD patients more likely to phenoconvert more rapidly. This could help with recruitment of participants in disease‐modifying therapies.

## 2 Subjects/Materials and Methods

### 2.1 Study Data

Participant data came from the Parkinson Progression Marker Initiative (PPMI), an ongoing longitudinal observational study, collecting samples as a biobank of PwP and HCs from North America and Europe. The goal of the PPMI is to encourage a collaborative effort of researchers to use data to identify markers of PD risk, diagnosis, and progression. More data on inclusion and exclusion for each cohort can be found on their website, www.ppmi‐info.org/study‐design. PPMI studies were approved by the institutional review boards at each of the participating sites, and written informed consent was obtained from participants prior to enrollment.

### 2.2 Study Population

Permission was obtained through the PPMI data portal at the Laboratory of Neuroimaging at the University of Southern California. The information was accessed on October 21, 2025. Data analyzed in this study included enrolled participants from April 1, 2014 to October 1, 2025. This study cohort included prodromal PD patients. Per PPMI guidelines, inclusion criteria for prodromal PD were age greater than 60 with RBD, hyposmia with DaTscan deficit, or a genetic risk variant (SNCA, LRRK2, and GBA).

### 2.3 Clinical and Biomarker Assessments

To evaluate the potential for the αSyn‐SAA, whole blood miR‐7‐5p and serum NDEV αSyn as biomarkers for phenoconversion, the following variables were collected: αSyn‐SAA data, whole blood miR‐7‐5p levels, serum NDEV αSyn levels, age, genetic status, sex, and Hoehn and Yahr Scale scores. Serum NDEV αSyn levels were obtained directly from the PPMI database. Briefly, serum was isolated and immunocapture was used to isolate L1CAM EVs from 250 μL of serum. Electrochemiluminescence was performed in 96‐well Meso Scale Discover U‐Plex plates. Antibody pairs for αSyn were provided by MSD and pre‐conjugated with biotin and ruthenium tag. The rabbit monoclonal antibody MJFR1 (Abcam) was used for capture. This binds to monomeric and aggregated forms of αSyn. A monoclonal mouse antibody was used for detection. NDEV αSyn concentration was measured in pg/mL. the natural log of NDEV αSyn (log[NDEV αSyn]) was used for normalization.

miRNA data were also obtained from the PPMI database. Whole blood was collected in PAXgene tubes and sequenced using an iIllumina NovaSeq6000 platform with Bioo Scientific smRNA library preparation. Expression files processed through PPMI were provided as raw counts and reads per million (RPM). For miRNAs, data were normalized to reads per million mapped to miRNAs (RPMMM), and miRNAs with zero expression (RPM = 0) in > 50% of samples were excluded. The natural log of miR‐7‐5p (log[miR‐7‐5p]) was used for normalization. Full details of sequencing and preprocessing are available in PPMI documentation and in the methods of a previous study [16]. While RNA sequencing was done in two phases, miRNA sequencing was done in the first phase (PPMI Project 133).

The αSyn‐SAA data were obtained from the PPMI database as well. The Amprion αSyn‐SAA was done as described previously [19].

For baseline characteristics, log[NDEV αSyn] levels, and age were presented as median (quartiles) and were compared between groups using a Mann–Whitney *U* test. Log[miR‐7‐5p] levels were presented as pooled mean (standard error) and were compared using a *t*‐test. Sex differences were presented as counts (percentages) and were compared using a chi‐squared test.

Cox proportional hazards regression analyses were performed to examine baseline log[miR‐7‐5p], log[NDEV αSyn], and the αSyn‐SAA versus time to development of PD. Both unadjusted and multivariable‐adjusted models were constructed. Adjusted models included age and sex as covariates.

Missing miR‐7‐5p data were imputed using the mice package in R. The predictive mean matching (PPM) method was used. Five imputations were run with 20 iterations for each imputed dataset. Convergence was assessed by visual inspection of trace plots generated from the mice package. There was stable log[miR‐7‐5p] level estimates across the five imputations. The cutpoints were calculated using the OptSurvCutR package in R. A log‐rank test was used to estimate the cutpoints. The R survival package was used for the Cox regression analysis using the Wald statistic to estimate *p* values for log[NDEV αSyn] and the αSyn‐SAA. For log[miR‐7‐5p] data, hazard ratios (HRs), confidence intervals and *p* values of imputed datasets were combined using Rubin's rules. To check whether the models violated assumptions for Cox regression analysis, the weighted residuals for the Cox regression analyses were analyzed using the survival package in R. A plot of scaled Schoenfeld residuals versus time was assessed. Cutpoints were verified using the OptSurvCutR R package. Cutpoints were verified using bootstrapping with 500 replicates. All statistical analyses were performed using R software, version 4.4.2. All tests were two‐sided, and statistical significance was defined as *p* < 0.05.

The age and sex distributions did not differ significantly between individuals missing data and those who were not missing data. The number of individuals with imputed whole blood miR‐7‐5p was 42. Serum log[NDEV αSyn] was not imputed in individuals missing data as too many individuals were missing these values to accurately impute the missing data. The variables in the imputed model included age, sex, baseline log[NDEV αSyn], baseline log[miR‐7‐5p], PD status, and an estimate of cumulative baseline hazard using the Nelson–Aalen estimator. A chi‐squared test showed that the distribution of genetic status was different between those with and those without log[NDEV αSyn] data (*p* = 0.001). This was mostly due to a higher proportion of individuals with GBA mutations in those with log[NDEV αSyn] data and a higher proportion of individuals without genetic mutations in those without log[NDEV αSyn] data. Although genetic status differed between those with and without log[NDEV αSyn] data, follow‐up time, frequencies of phenoconversion and baseline log[miR‐7‐5p] levels did not differ between those with and without log[NDEV αSyn] data. This suggests that those missing log[NDEV αSyn] data did not strongly affect the primary study outcome.

## 3 Results

Among 313 patients with either miR‐7‐5p or NDEV αSyn levels available, 79 (25.2%) developed PD during follow‐up. Among 101 patients with NDEV αSyn baseline levels available, 18 patients developed PD (17.8%). The cohort was composed of 59 without genetic mutations (18.85%), 87 with GBA mutations (27.8%), 156 with LRRK2 mutations (49.84%), six with SNCA mutations (1.92%), and five without genetic information (1.6%).

### 3.1 Baseline Characteristics

Baseline characteristics according to phenoconversion status can be found in Table 1. Patients who developed PD had significantly higher baseline log[miR‐7–5p] levels compared with those who did not (pooled *p* < 0.001, Table 1). They were also older (*p* = 0.01). Sex distribution and baseline log[NDEV αSyn] levels did not differ significantly between those who developed PD and those who did not.

**TABLE 1:** Baseline characteristics according to phenoconversion status.

Characteristics	PD	At risk	*p*	All
Log[miR‐7‐5p], pooled mean (SE)^a^	3.36 (0.09)	2.97 (0.06)	< 0.001	3.07 (0.04)
Log[NDEV αSyn], median (quartiles)^b^	3.44 (3.12, 4.10)	3.05 (2.61, 3.85)	0.14	3.27 (2.63, 3.89)
Age, median (quartiles)	65.1 (60.5, 67.7)	61.9 (56.6, 67.6)	0.01	63 (57.7, 67.65)
Male/female (ratio)	42/37 (1.14)	100/134 (0.75)	0.11	142/171

Abbreviations: NDEV αSyn, neuron‐derived extracellular vesicle α‐synuclein; PD, Parkinson's disease.

^a^ PD, 79; at risk, 234.

^b^ PD, 18; at risk, 83.

### 3.2 Cox Proportional Hazards Analysis

Biomarkers were analyzed as continuous and categorical variables using data‐derived cutoffs. Log‐rank analysis showed a cutoff for log[miR‐7‐5p] of ≥ 3.31 (*p* < 0.001) and a cutoff for log[NDEV αSyn] of ≥ 2.91 (*p* = 0.01). The corresponding exponentiated cutoffs were 27.39 RPM mapped for miR‐7‐5p and 18.36 pg/mL for NDEV αSyn. HRs with 95% CIs were reported. Bootstrapping analysis was done using the OptSurvCutR R package. There were 500 replicates done and the optimal cutpoints for log[mir‐7‐5p] was 3.31 and the optimal cutpoint for log[NDEV αSyn] was 2.91. The median cutpoints for log[miR‐7‐5p] were 3.18 (95% CI, 2.44–4.63), 3.31 (95% CI, 2.37–4.62), 3.32 (95% CI, 2.35–4.38), 3.31 (95% CI, 2.37–4.70), and 3.32 (95% CI, 2.62–4.69) for each of the imputed datasets with bootstrap resampling. The median of these cutpoints was calculated as 3.31. The median cutpoint for log[NDEV αSyn] was 2.91 (95% CI, 2.21–4.74) with bootstrap resampling. However, both the log[miR‐7‐5p] and log[NDEV αSyn] cutpoints showed substantial variability in selected thresholds, indicating the cutpoints are unstable. The thresholds should be considered exploratory and hypothesis‐generating and require validation in an independent cohort. Density plots of the 500 individual bootstrap cutpoints for each analysis can be found in Supporting Information Materials (Figures S1 and S2).

Cox.zph tests and plots of scaled Schoenfeld residuals versus time were done on SAA αSyn, log[miR‐7‐5p] and log[NDEV αSyn] Cox models with and without covariates as well as on the covariates themselves. Cox.zph tests and plots were also done on each imputation in Cox models with miR‐7‐5p. The results of these tests are available in Supporting Information Materials (Table S1). Using the cox.zph test, we found that in a Cox model with log[NDEV αSyn], age, and sex, age had a significant *p* value. On review of a spline plot of the residuals, there does not appear to be a linear relationship between time and residuals (Figure S16). The correlation came up due to the small sample size (*N* = 18). Also, in a model with positive αSyn‐SAA and miR‐7‐5p cutpoint above threshold, age, and sex, one of the imputations showed a significant *p* value above threshold. On review of the plot, there does not appear to be a clear linear relationship between residuals and time. Also, none of the other imputations showed a trend (Figure S25). The full results of the cox.zph tests can be found in the Supporting Information Materials (Table S1) along with the plots of residuals versus time (Figures S3–S26). The rest of the cox.zph tests did not show time‐dependent effects.

### 3.3 Whole Blood miR‐7–5p

**FIGURE 1:**
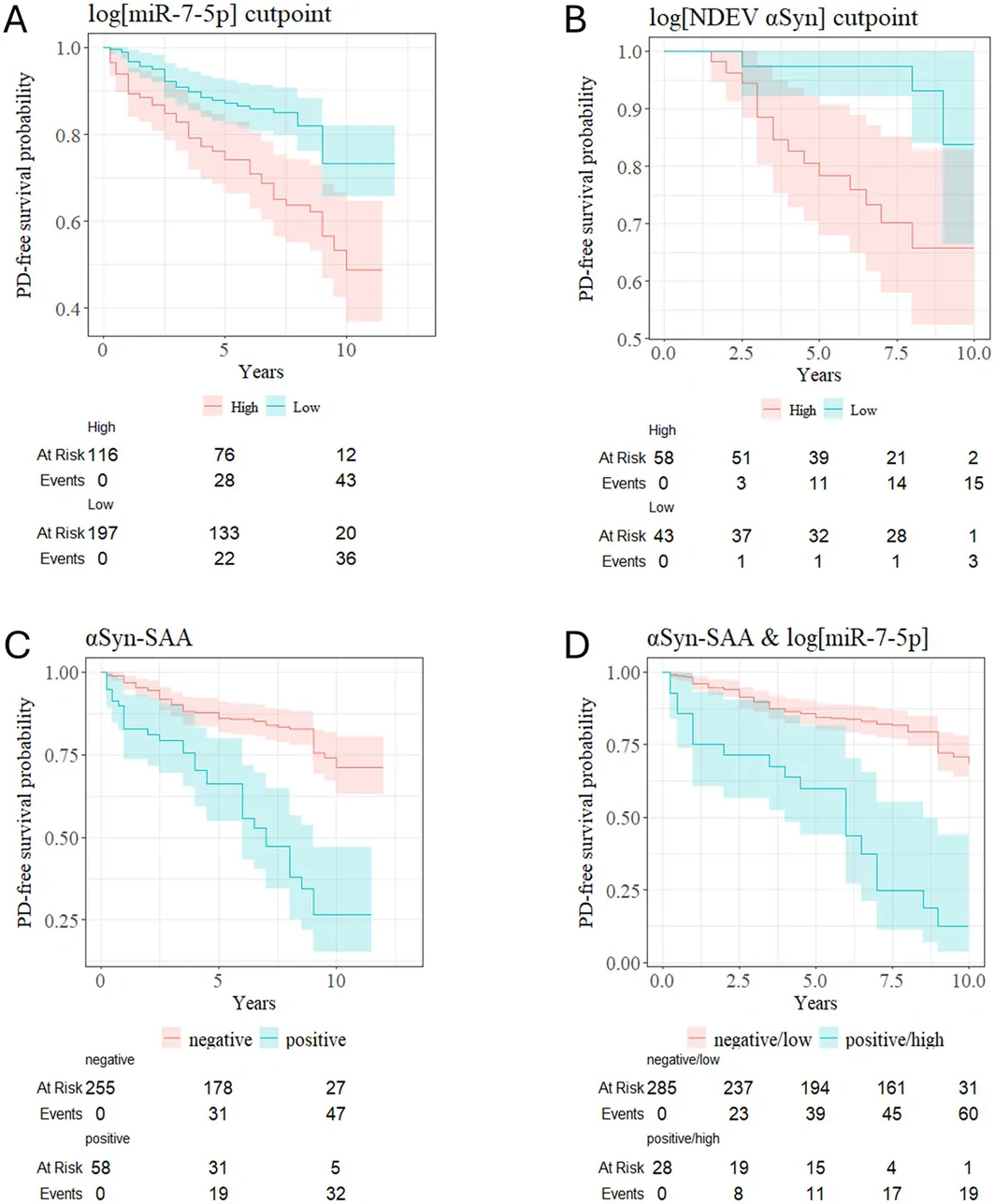
Survival curves for phenoconversion of prodromal Parkinson's disease patients based on data‐derived cutpoints. (A) The red line shows PD‐free survival probabilities in individuals with baseline log[miR‐7‐5p] above threshold while the blue line shows survival probabilities in those with log[miR‐7‐5p] below threshold. The data is representative from one of the five imputed datasets for log[miR‐7‐5p]. (B) The red line shows PD‐free survival probabilities versus time in individuals with baseline log[NDEV αSyn] above threshold while the blue line shows survival probabilities in those with log[NDEV αSyn] below threshold. (C) The blue line shows PD‐free survival probabilities in individuals with a positive αSyn‐SAA at baseline while the red line shows survival probabilities in individuals with a negative αSyn‐SAA at baseline. (D) The blue line shows PD‐free survival probabilities in individuals with both positive baseline αSyn‐SAA and log[miR‐7‐5p] above threshold while the red line shows survival probabilities in individuals without both positive baseline αSyn‐SAA and log[miR‐7‐5] above threshold. The data is representative from one of the five imputed datasets for log[miR‐7‐5p]. PD, Parkinson's disease; NDEV αSyn, neuron‐derived α‐synuclein; αSyn‐SAA, α‐synuclein seed amplification assay.

In unadjusted Cox regression analysis with pooled HRs, for each unit increase in baseline log[miR‐7–5p], there is 1.60 times increased PD phenoconversion (pooled *p* = < 0.001) (Table 2). A representative survival curve for one of the miR‐7‐5p imputations is shown (Figure 1A). The pooled HRs remained significant after adjustment for age and sex with 1.57 times increased PD phenoconversion for each unit increase in log[miR‐7‐5p] (pooled *p* = 0.003). When analyzed categorically, pooled HRs for elevated log[miR‐7–5p] was associated with 2.23 times increased PD phenoconversion in an unadjusted model (95% CI, 1.42–3.49, pooled *p* < 0.001) and 2.19 times increased PD phenoconversion in an adjusted model (95% CI, 1.37–3.5, pooled *p* = 0.001). Increasing age was associated with 1.05 times increased PD phenoconversion (95% CI, 1.02–1.08; pooled *p* = 0.003), whereas sex was not associated with increased PD phenoconversion.

**TABLE 2:** Association between baseline log[miR‐7–5p] levels and risk of phenoconversion in Cox proportional hazards models.

Variable	Unadjusted pooled HR (95% CI)	*p*	Adjusted pooled HR (95% CI)	*p*
Continuous model				
Log[miR‐7‐5p]	1.60 (1.22–2.10)	< 0.001	1.57 (1.17–2.10)	0.003
Age (per year)	—		1.05 (1.02–1.09)	0.003
Sex	—		1.11 (0.68–1.81)	0.69
Categorical model				
Log[miR‐7‐5p] > 3.31	2.23 (1.42–3.49)	< 0.001	2.19 (1.37–3.5)	0.001
Age (per year)	—	—	1.05 (1.02–1.09)	0.002
Sex	—	—	1.06 (0.66–1.69)	0.818

Abbreviations: CI, confidence interval; HR, hazard ratio; PD, Parkinson disease.

### 3.4 NDEV αSyn

Continuous NDEV αSyn levels were not significantly associated with PD phenoconversion in either unadjusted or adjusted analyses (Table 3). However, when dichotomized, elevated log[NDEV αSyn] levels were associated with 4.39 times increased phenoconversion in unadjusted (95% CI, 1.26–15.25, *p* = 0.02) and 4.46 times increased phenoconversion in adjusted models (95% CI, 1.26–15.79, *p* = 0.02) (Figure 1B). Age remained independently associated with 1.05 times increase PD phenoconversion (95% CI, 1.00–1.14; *p* = 0.04), while sex was not associated with increased phenoconversion.

**TABLE 3:** Association between baseline log[NDEV αSyn] levels and risk of PD conversion in Cox proportional hazards models.

Variable	Unadjusted HR (95% CI)	Wald *p*	Adjusted HR (95% CI)	Wald *p*
Continuous model				
Log[NDEV αSyn]	1.43 (0.85–2.40)	0.18	1.42 (0.84–2.40)	0.20
Age (per year)	—		1.07 (1.00–1.14)	0.04
Sex	—		0.74 (0.26–2.09)	0.6
Categorical model				
Log[NDEV αSyn] > 2.91	4.39 (1.26–15.25)	0.02	4.46 (1.26–15.79)	0.02
Age (per year)	—	—	1.05 (1.00–1.14)	0.04
Sex	—	—	0.58 (0.20–1.64)	0.30

Abbreviations: CI, confidence interval; HR, hazard ratio; NDEV αSyn, neuron‐derived α‐synuclein; PD, Parkinson disease.

### 3.5 αSyn‐SAA

Participants with positive αSyn‐SAA had 4.13 times higher risk of phenoconversion in unadjusted (95% CI, 2.56–6.65, *p* < 0.001) and 3.62 times increased risk in adjusted models (95% CI, 2.21–5.93, *p* < 0.001) (Figure 1C). Baseline age was associated with PD phenoconversion (adjusted HR per year, 1.05 [95% CI, 1.01–1.08]; *p* = 0.009), whereas sex was not (Tables 4 and 5).

**TABLE 4:** Association between baseline αSyn‐SAA and risk of PD phenoconversion in Cox proportional hazards models.

Variable	Unadjusted HR (95% CI)	Wald *p*	Adjusted HR (95% CI)	Wald *p*
αSyn‐SAA	4.13 (2.56–6.65)	< 0.001	3.62 (2.21–5.93)	< 0.001
Age	—	—	1.05 (1.01–1.08)	0.009
Sex	—	—	1.04 (0.64–1.69)	0.88

Abbreviations: CI, confidence interval; HR, hazard ratio; PD, Parkinson disease, αSyn‐SAA, α‐synuclein seed amplification assay.

**TABLE 5:** Association between combined αSyn‐SAA and log[miR‐7‐5p] levels above its threshold on risk of PD phenoconversion in Cox proportional hazards models.

Variable	Unadjusted pooled HR (95% CI)	*p*	Adjusted pooled HR (95% CI)	*p*
αSyn‐SAA positive and Log[mir7‐5p] > 3.31	4.72 (2.72–8.19)	< 0.001	4.31 (2.46–7.53)	< 0.001
Age (per year)	—	—	1.06 (1.022–1.096)	0.002
Sex	—	—	1.09 (0.67–1.77)	0.72

Abbreviations: CI, confidence interval; HR, hazard ratio; PD, Parkinson disease, αSyn‐SAA, α‐synuclein seed amplification assay.

### 3.6 Whole Blood miR‐7‐5p and αSyn‐SAA

Individuals with both positive αSyn‐SAA and log[miR‐7‐5p] levels above threshold had 4.72 times increased phenoconversion in an unadjusted model (95% CI, 2.72–8.19, *p* < 0.001) and 4.31 times increased risk in an adjusted model (95% CI, 2.46–7.53, *p* < 0.001). A representative survival curve for one of the miR‐7‐5p imputations combined with αSyn‐SAA data is shown (Figure 1D). Baseline age was associated with PD phenoconversion (adjusted HR per year, 1.06 [95% CI, 1.02–1.10]; *p* = 0.002), but sex was not.

## 4 Discussion

Higher miR‐7‐5p were associated with increased PD phenoconversion when age and sex were included in Cox regression model as a continuous or categorical variable. On the other hand, higher NDEV αSyn levels were associated with increased phenoconversion as a categorical variable but not as a continuous variable. A positive αSyn‐SAA was associated with increased phenoconversion as well. Combining both a positive αSyn‐SAA and log[miR‐7‐5p] above threshold resulted in a higher risk of phenoconversion than either test individually.

### 4.1 The Diagnostic Imperative

The clinical and therapeutic management of PD is fundamentally constrained by a pronounced diagnostic lag. Current clinical diagnostic frameworks rely heavily on the manifestation of hallmark motor dysfunction; however, by the time these signs emerge, it is estimated that 30% to 50% of the dopaminergic neurons in the substantia nigra have already been irreversibly lost [20]. This pathophysiological reality essentially dooms many neuroprotective interventions to failure by initiating treatment after critical neuronal infrastructure is destroyed. The central objective of this retrospective analysis of the PPMI database was to evaluate the predictive utility of blood miR‐7‐5p, NDEV αSyn, and the αSyn‐SAA to bridge this diagnostic gap.

### 4.2 The Molecular Pathogenesis: The αSyn and miR‐7‐5p Axis

The strategic selection of miR‐7‐5p, the αSyn‐SAA, and NDEV αSyn is predicated on their critical and intertwined involvement in the αSyn‐neuroinflammation axis of PD pathology. Pathologically, PD is characterized by the toxic aggregation of αSyn in neurons, which directly drives regional neuronal degeneration and reciprocal neuroinflammatory responses. Within this complex regulatory environment, miR‐7‐5p acts as a multifaceted homeostatic master regulator. Extensive literature corroborates that miR‐7‐5p directly represses αSyn expression and mitigates the toxicity induced by its aggregation [13, 15, 21]. Furthermore, miR‐7‐5p facilitates the clearance of these existing toxic aggregates by promoting the autophagy‐lysosome pathway, and it actively suppresses neuroinflammation by targeting the NLRP3 inflammasome [11, 12, 22].

A previous study found miR‐7‐5p was downregulated in the substantia nigra of established PD patients [15]. miR‐7‐5p also decreased over time in the blood of PwP [17]. However, our transcriptomic data robustly supports the recent work of Li et al. (2024) [18], revealing that miR‐7‐5p is upregulated in the blood of prodromal patients who experience a more rapid trajectory toward phenoconversion. It is likely that much of the increase in blood miR‐7‐5p is from EVs based on the study by Li et al. miR‐7‐5p is likely initially upregulated as a compensatory, neuroprotective response in prodromal PD patients. However, pesticides can reduce levels of miR‐7‐5p potentially inhibiting this compensatory response [23, 24].

### 4.3 Feasibility of Liquid Biopsies: Blood vs. CSF

The evolution of “liquid biopsies” marks a fundamental shift in clinical feasibility, specifically moving away from invasive CSF sampling toward serum‐based transcriptomics. While CSF remains a proximal reflection of brain chemistry, the requirement for serial lumbar punctures presents a high patient burden, limiting its utility in the frequent, longitudinal monitoring required for large‐scale observational studies like the PPMI.

### 4.4 Targeted NDEV Isolation

EVs are cell‐derived membranous particles essential for molecular trafficking and the elimination of unwanted proteins. Because EVs carry the specific protein and miRNA “fingerprints” of their parental cells, and their lipid bilayer protects internal cargo from circulating RNases and proteases, they are diagnostically highly relevant. In this study, NDEVs were extracted from a mere 250 μL of serum using immunocapture targeted at the L1CAM marker, a transmembrane protein acting as a specific fingerprint for neuronal origin. Crucially, the MJFR1 antibody uniquely binds to both monomeric and aggregated forms of αSyn, ensuring a comprehensive assessment of the total pathological protein load [25]. This provides a direct, non‐invasive window into the brain.

Nanoparticle analysis has shown that L1CAM pulls down particles in the size range of EVs [26]. Further, western blots have shown that these EVs have other neuronal markers, such as beta‐tubulin and EV markers, such as Alix and CD81 [26]. Also, confocal microscopy has shown that L1CAM and colocalizes with CD81, CD63 and Alix on microbeads [26]. Further, nanoscale flow cytometry shows L1CAM particles around 100–200 nm, consistent with EVs [26].

### 4.5 Whole Blood Transcriptomics

Conversely, the miR‐7‐5p data was obtained from whole blood providing a broader, systemic transcriptomic profile. As whole blood was used, the measurements reflect both intracellular and extracellular concentrations of miR‐7‐5p. By combining standardized whole blood collection for systemic miRNAs with L1CAM‐isolated EVs for brain‐specific proteins, researchers can leverage the best of both methodologies to bypass the need for invasive lumbar punctures. Furthermore, the combined predictive ability of the αSyn‐SAA and blood miR‐7‐5p appeared to be better than the αSyn‐SAA alone in identifying individuals more likely to phenoconvert. This is largely likely due to the large number of LRRK2+ and GBA+ patients in this prodromal cohort. A previous study found that while 86% of sporadic prodromal PD patients had positive αSyn‐SAAs, only 28% of GBA, 20% of LRRK2, and 0% of SNCA positive PwP had positive αSyn‐SAAs indicating αSyn‐SAA alone is not ideal for identifying individuals more likely to phenoconvert in these populations [7].

### 4.6 Validation Scale: The PPMI Database vs. Smaller Independent Cohorts

A major methodological strength of this analysis is its foundation in the PPMI database. The PPMI is a massive, multi‐center longitudinal biobank designed to encourage the collaborative identification of PD markers. Compared to earlier independent studies exploring EV biomarkers, such as Li et al., which often rely on smaller sample sizes, the PPMI provides a much larger and more statistically robust platform for validation.

Our initial evaluation pool included 313 prodromal patients with baseline miR‐7‐5p levels, of which 79 (25.2%) developed PD during follow‐up. For NDEV αSyn baseline levels, 101 patients were available, with 18 (17.8%) phenoconverting. However, it is vital to acknowledge the sample limitations inherent in novel biomarker aggregation. When combining both novel markers for the joint multivariable model, the sample size was restricted to the 101 patients who possessed both measurements simultaneously. This reduced subset size is likely the primary reason why significance for continuous variables was not reached in the combined model, highlighting the immense difficulty of harmonizing multiple novel transcriptomic and proteomic datasets.

### 4.7 Categorical vs. Continuous Divergence

A fascinating statistical divergence occurred between continuous and categorical modeling. For instance, continuous baseline log[NDEV αSyn] was not significantly associated with PD phenoconversion in adjusted analyses (HR 1.42, *p* = 0.20). However, when dichotomized using the mathematically derived 2.91 cutpoint, elevated levels became highly significant (adjusted HR 4.46, *p* = 0.02). This suggests that pathological progression may not scale linearly with biomarker concentration; rather, there may exist a biological “threshold” or tipping point that, once breached, signals imminent motor decline. Alternatively, the difference between continuous and categorical results may be due to unstable threshold selection and overfitting. There needs to be independent validation of the results in future studies.

### 4.8 Prodromal Phenotypes: Decoupling RBD, Hyposmia, and Genetics

The prodromal phase of PD is defined by a constellation of clinical and etiological risk factors that can precede motor decline by several years. The cohorts evaluated in this study were carefully selected based on specific PPMI inclusion criteria for individuals over age 60, requiring the presence of either REM sleep behavior disorder (RBD), hyposmia coupled with a DaTscan deficit, or a known genetic risk variant such as SNCA, LRRK2, or GBA.

When these established clinical phenotypes are paired with elevated baseline log miR‐7‐5p and NDEV αSyn, they define a highly accurate “fast‐track” phenotype. However, it is necessary to distinguish clinical anchors from etiological genetic risk factors; while genetic markers establish a lifetime biological predisposition, they frequently lack the temporal precision required to predict the exact timing of phenoconversion. Blood‐based EVs bridge this temporal gap by reflecting active, real‐time pathogenesis.

Sample sizes were too restrictive for a full subgroup breakdown. As LRRK2 mutations have been found to heighten inflammatory responses of microglia, miR‐7‐5p may be more affected in LRRK2 patients as opposed to idiopathic PD patients. Future research will be needed to determine whether miR‐7‐5p is more affected in LRRK2 patients as opposed to idiopathic PD patients.

### 4.9 Strategic Implications: Trial Enrichment for Disease‐Modifying Therapies

The goal of discovering robust phenoconversion biomarkers is the strategic optimization of clinical trials. The inclusion of prodromal patients who possess a low immediate risk of phenoconversion introduces massive statistical “noise” into longitudinal datasets. This noise masks potential early therapeutic effects, leading to false negatives and necessitating prohibitively large, expensive sample sizes.

By deploying the combined biomarker model (both miR‐7‐5p above its threshold and positive αSyn‐SAA, HR 4.31), trial designers can effectively filter out slow progressors. Enriching trials with these precisely selected participants allows researchers to isolate the most aggressive forms of the disease, drastically increasing the statistical power to detect neuroprotective signals during Phase II and III trials.

### 4.10 Global Burden

The imperative for predictive precision medicine in PD cannot be overstated. PD is the fastest‐growing neurological disorder on the planet. Current projections forecast an alarming 76% increase in PD prevalence by the year 2050, surging to approximately 267 cases per 100,000 individuals globally. This epidemiological trajectory implies an unprecedented and staggering economic and psychological burden on caregivers and global healthcare systems alike.

By combining the standardized collection of systemic whole‐blood miR‐7‐5p with the highly targeted L1CAM immunocapture of NDEV αSyn, researchers can non‐invasively monitor the collapse of protein homeostasis and the escalation of neuroinflammation. As blood‐based testing replaces the procedural burdens of serial lumbar punctures, it significantly lowers the barrier to trial participation and enables high‐frequency longitudinal screening. Moving forward, the integration of these EV‐based methodologies will be instrumental in closing the diagnostic gap, allowing the field of neurology to move beyond the era of reactive symptomatic management and usher in a new paradigm of proactive, biologically targeted neuroprotection before widespread neuronal devastation occurs.

## 5 Conclusion

This study found that the combinations of whole blood miR‐7‐5p and αSyn‐SAA could help enrich for prodromal PD patients more likely to phenoconvert sooner. We corroborated previous studies finding NDEV αSyn could also be used to enrich for prodromal PD patients more likely to phenoconvert sooner. Given the small number of individuals with NDEV αSyn, the results are more exploratory and hypothesis generating. A larger cohort is needed to confirm NDEV αSyn as a marker that could be used to enrich for individuals more likely to phenoconvert. In the future, with a larger cohort of individuals with both blood miR‐7‐5p levels and NDEV αSyn levels, whether the combination of these markers as well could further enrich for individuals more likely to phenoconvert could be studied.

## Author Contributions

**Shayan Zadegan:** investigation, writing – original draft, methodology, visualization, writing – review and editing, formal analysis, data curation. **Praveen Velammal:** methodology, validation, visualization, writing – review and editing, formal analysis, data curation, writing – original draft. **Kanmani Muthiah:** writing – review and editing, writing – original draft, data curation. **Madhu Jatty:** writing – review and editing. **Christopher Adams:** conceptualization, methodology, visualization, writing – review and editing, writing – original draft, formal analysis, data curation, supervision.

## Funding

The authors have nothing to report.

## Conflicts of Interest

The authors declare no conflicts of interest.

## Supporting information


**Figure S1:** Bootstrap distributions for log[miR‐7‐5p] cutpoints for imputations 1 (A), 2 (B), 3 (C), 4 (D), and 5 (E).
**Figure S2:** Bootstrap distribution for log[NDEV α‐syn] cutpoints.
**Figure S3:** Splines of residuals versus time for a Cox model with log[miR‐7‐5p] as a continuous variable.
**Figure S4:** Spline residuals versus time for a Cox model with log[miR‐7‐5p] as a dichotomous variable.
**Figure S5:** Spline residuals versus time for a Cox model with log[miR‐7‐5p] (A), baseline age (B), and sex (C) for the first imputation of log[miR‐7‐5p].
**Figure S6:** Spline residuals versus time for a Cox model with log[miR‐7‐5p] (A), baseline age (B), and sex (C) for the second imputation of log[miR‐7‐5p].
**Figure S7:** Spline residuals versus time for a Cox model with log[miR‐7‐5p] (A), baseline age (B), and sex (C) for the third imputation of log[miR‐7‐5p].
**Figure S8:** Spline residuals versus time for a Cox model with log[miR‐7‐5p] (A), baseline age (B), and sex (C) for the fourth imputation of log[miR‐7‐5p].
**Figure S9:** Spline residuals versus time for a Cox model with log[miR‐7‐5p] (A), baseline age (B), and sex (C) for the fifth imputation of log[miR‐7‐5p].
**Figure S10:** Spline residuals versus time for a Cox model with log[miR‐7‐5p] cutpoint (A), baseline age (B), and sex (C) for the first imputation of log[miR‐7‐5p].
**Figure S11:** Spline residuals versus time for a Cox model with log[miR‐7‐5p] cutpoint (A), baseline age (B), and sex (C) for the second imputation of log[miR‐7‐5p].
**Figure S12:** Spline residuals versus time for a Cox model with log[miR‐7‐5p] cutpoint (A), baseline age (B), and sex (C) for the third imputation of log[miR‐7‐5p].
**Figure S13:** Spline residuals versus time for a Cox model with log[miR‐7‐5p] cutpoint (A), baseline age (B), and sex (C) for the fourth imputation of log[miR‐7‐5p].
**Figure S14:** Spline residuals versus time for a Cox model with log[miR‐7‐5p] cutpoint (A), baseline age (B), and sex (C) for the fifth imputation of log[miR‐7‐5p].
**Figure S15:** Spline residuals versus time for a Cox models with log[NDEV α‐syn] (A) and log[NDEV α‐syn] cutpoint (B).
**Figure S16:** Spline of residuals versus time for a Cox model with log[NDEV α‐syn] (A), baseline age (B), and sex (C).
**Figure S17:** Spline of residuals versus time for a Cox model with log[NDEV α‐syn] cutpoint (A), baseline age (B), and sex (C).
**Figure S18:** Spline of residuals versus time for a Cox model with CSF α‐syn seed amplification assay (SAA).
**Figure S19:** Spline of residuals versus time for Cox model with CSF α‐syn seed amplification assay (SAA) (A), baseline age (B), and sex (C).
**Figure S20:** Spline of residuals versus time for a Cox models with combined CSF α‐syn seed amplification assay (SAA) and log[miR‐7‐5p] cutpoint in log[miR‐7‐5p] imputations 1 (A), 2 (B), 3 (C), 4 (D), and 5 (E).
**Figure S21:** Spline of residuals versus time for a Cox model with combined CSF α‐syn seed amplification assay (SAA) and log[miR‐7‐5p] cutpoint, baseline age, and sex using data from log[miR‐7‐5p] imputation 1.
**Figure S22:** Spline of residuals versus time for a Cox model with combined CSF α‐syn seed amplification assay (SAA) and log[miR‐7‐5p] cutpoint, baseline age, and sex using data from log[miR‐7‐5p] imputation 2.
**Figure S23:** Spline of residuals versus time for a Cox model with combined CSF α‐syn seed amplification assay (SAA) and log[miR‐7‐5p] cutpoint, baseline age, and sex using data from log[miR‐7‐5p] imputation 3.
**Figure S24:** Spline of residuals versus time for a Cox model with combined CSF α‐syn seed amplification assay (SAA) and log[miR‐7‐5p] cutpoint, baseline age, and sex using data from log[miR‐7‐5p] imputation 4.
**Figure S25:** Spline of residuals versus time for a Cox model with combined CSF α‐syn seed amplification assay (SAA) and log[miR‐7‐5p] cutpoint, baseline age, and sex using data from log[miR‐7‐5p] imputation 5.
**Figure S26:** Spline of residuals versus time for Cox models with sex (A) and age (B).

**Table S1:** Cox.zph tests for each Cox model in the analyses.

## Data Availability

Data used in the preparation of this article were obtained from the Parkinson's Progression Markers Initiative (PPMI) database (www.ppmi‐info.org/access‐data‐specimens/download‐data). For up‐to‐date information on the study, visit ppmi‐info.org.
