# Supplementary material for: microRNA‐7‐5p and α‐Synuclein SAA Predict Parkinson's Disease Phenoconversion
Source: Ann Clin Transl Neurol. 2026 Sep 25:10.1002/acn3.70539. Online ahead of print. doi: 10.1002/acn3.70539 (PMC13614880; doi:10.1002/acn3.70539)
Supplement: Supplementary file 1 [file ACN3-9999-0-s001.docx]

Supplementary figures

Figure S1. Bootstrap distributions for log[miR-7-5p] cutpoints for imputations 1 (A), 2 (B), 3 (C), 4 (D), and 5 (E).

Figure S2. Bootstrap distribution for log[NDEV α-syn] cutpoints.

Figure S3. Splines of residuals versus time for a Cox model with log[miR-7-5p] as a continuous variable.

Figure S4. Spline residuals versus time for a Cox model with log[miR-7-5p] as a dichotomous variable.

Figure S5. Spline residuals versus time for a Cox model with log[miR-7-5p] (A), baseline age (B), and sex (C) for the first imputation of log[miR-7-5p].

Figure S6. Spline residuals versus time for a Cox model with log[miR-7-5p] (A), baseline age (B), and sex (C) for the second imputation of log[miR-7-5p].

Figure S7. Spline residuals versus time for a Cox model with log[miR-7-5p] (A), baseline age (B), and sex (C) for the third imputation of log[miR-7-5p].

Figure S8. Spline residuals versus time for a Cox model with log[miR-7-5p] (A), baseline age (B), and sex (C) for the fourth imputation of log[miR-7-5p].

Figure S9. Spline residuals versus time for a Cox model with log[miR-7-5p] (A), baseline age (B), and sex (C) for the fifth imputation of log[miR-7-5p].

Figure S10. Spline residuals versus time for a Cox model with log[miR-7-5p] cutpoint (A), baseline age (B), and sex (C) for the first imputation of log[miR-7-5p].

Figure S11. Spline residuals versus time for a Cox model with log[miR-7-5p] cutpoint (A), baseline age (B), and sex (C) for the second imputation of log[miR-7-5p].

Figure S12. Spline residuals versus time for a Cox model with log[miR-7-5p] cutpoint (A), baseline age (B), and sex (C) for the third imputation of log[miR-7-5p].

Figure S13. Spline residuals versus time for a Cox model with log[miR-7-5p] cutpoint (A), baseline age (B), and sex (C) for the fourth imputation of log[miR-7-5p].

Figure S14. Spline residuals versus time for a Cox model with log[miR-7-5p] cutpoint (A), baseline age (B), and sex (C) for the fifth imputation of log[miR-7-5p].

Figure S15. Spline residuals versus time for a Cox models with log[NDEV α-syn] (A) and log[NDEV α-syn] cutpoint (B).

Figures S16. Spline of residuals versus time for a Cox model with log[NDEV α-syn] (A), baseline age (B), and sex (C).

Figures S17. Spline of residuals versus time for a Cox model with log[NDEV α-syn] cutpoint (A), baseline age (B), and sex (C).

Figure S18. Spline of residuals versus time for a Cox model with CSF α-syn seed amplification assay (SAA).

Figure S19. Spline of residuals versus time for Cox model with CSF α-syn seed amplification assay (SAA) (A), baseline age (B), and sex (C).

Figure S20. Spline of residuals versus time for a Cox models with combined CSF α-syn seed amplification assay (SAA) and log[miR-7-5p] cutpoint in log[miR-7-5p] imputations 1 (A), 2 (B), 3 (C), 4 (D), and 5 (E).

Figure S21. Spline of residuals versus time for a Cox model with combined CSF α-syn seed amplification assay (SAA) and log[miR-7-5p] cutpoint, baseline age, and sex using data from log[miR-7-5p] imputation 1.

Figure S22. Spline of residuals versus time for a Cox model with combined CSF α-syn seed amplification assay (SAA) and log[miR-7-5p] cutpoint, baseline age, and sex using data from log[miR-7-5p] imputation 2.

Figure S23. Spline of residuals versus time for a Cox model with combined CSF α-syn seed amplification assay (SAA) and log[miR-7-5p] cutpoint, baseline age, and sex using data from log[miR-7-5p] imputation 3.

Figure S24. Spline of residuals versus time for a Cox model with combined CSF α-syn seed amplification assay (SAA) and log[miR-7-5p] cutpoint, baseline age, and sex using data from log[miR-7-5p] imputation 4.

Figure S25. Spline of residuals versus time for a Cox model with combined CSF α-syn seed amplification assay (SAA) and log[miR-7-5p] cutpoint, baseline age, and sex using data from log[miR-7-5p] imputation 5.

Figure S26. Spline of residuals versus time for Cox models with sex (A) and age (B).
