# Supplementary material for: microRNA‐7‐5p and α‐Synuclein SAA Predict Parkinson's Disease Phenoconversion
Source: Ann Clin Transl Neurol. 2026 Sep 25:10.1002/acn3.70539. Online ahead of print. doi: 10.1002/acn3.70539 (PMC13614880; doi:10.1002/acn3.70539)
Supplement: Supplementary file 2 [file ACN3-9999-0-s002.docx]

Supplementary table

Table S1. Cox.zph tests for each Cox model in the analyses.

| **Cox model** | **Chi-square** | **p-value** |
| --- | --- | --- |
| **Log[miR7-5p]** |  |  |
| Imputation 1 | 0.22 | 0.64 |
| Imputation 2 | 0.08 | 0.77 |
| Imputation 3 | 0.09 | 0.76 |
| Imputation 4 | 0.07 | 0.79 |
| Imputation 5 | 0.19 | 0.66 |
| **Log[miR7-5p] cutpoint** |  |  |
| Imputation 1 | 0.12 | 0.73 |
| Imputation 2 | 0.07 | 0.79 |
| Imputation 3 | 0.14 | 0.71 |
| Imputation 4 | 0.004 | 0.95 |
| Imputation 5 | 0.01 | 0.9 |
| **Log[miR7-5p] + Age + Sex** |  |  |
| **Imputation 1** |  |  |
| Log[miR-7-5p] | 0.28 | 0.6 |
| Age | 0.30 | 0.58 |
| Sex | 1.61 | 0.21 |
| Global | 1.77 | 0.62 |
| **Imputation 2** |  |  |
| Log[miR-7-5p] | 0.13 | 0.72 |
| Age | 0.33 | 0.56 |
| Sex | 1.42 | 0.23 |
| Global | 1.57 | 0.67 |
| **Imputation 3** |  |  |
| Log[miR-7-5p] | 0.11 | 0.75 |
| Age | 0.28 | 0.60 |
| Sex | 1.43 | 0.23 |
| Global | 1.59 | 0.66 |
| **Imputation 4** |  |  |
| Log[miR-7-5p] | 0.12 | 0.73 |
| Age | 0.33 | 0.57 |
| Sex | 1.53 | 0.22 |
| Global | 1.66 | 0.64 |
| **Imputation 5** |  |  |
| Log[miR-7-5p] | 0.07 | 0.79 |
| Age | 0.46 | 0.50 |
| Sex | 1.54 | 0.22 |
| Global | 1.85 | 0.60 |
| **miR-7-5p cutpoint + Age + Sex** |  |  |
| **Imputation 1** |  |  |
| miR-7-5p cutpoint | 0.17 | 0.68 |
| Age | 0.32 | 0.57 |
| Sex | 1.59 | 0.21 |
| Global | 1.75 | 0.63 |
| **Imputation 2** |  |  |
| miR-7-5p cutpoint | 0.10 | 0.75 |
| Age | 0.33 | 0.56 |
| Sex | 1.43 | 0.23 |
| Global | 1.59 | 0.66 |
| **Imputation 3** |  |  |
| miR-7-5p cutpoint | 0.19 | 0.66 |
| Age | 0.34 | 0.56 |
| Sex | 1.57 | 0.21 |
| Global | 1.74 | 0.63 |
| **Imputation 4** |  |  |
| miR-7-5p cutpoint | 0.02 | 0.89 |
| Age | 0.35 | 0.55 |
| Sex | 1.55 | 0.21 |
| Global | 1.72 | 0.63 |
| **Imputation 5** |  |  |
| miR-7-5p cutpoint | 0.007 | 0.93 |
| Age | 0.32 | 0.57 |
| Sex | 1.60 | 0.21 |
| Global | 1.94 | 0.59 |
| **Log[NDEV a-syn]** | 0.74 | 0.39 |
| **NDEV a-syn cutpoint** | 1 | 0.32 |
| **Log[NDEV a-syn] + Age + Sex** |  |  |
| Log[NDEV a-syn] | 0.83 | 0.36 |
| Age | 4.0 | **0.045** |
| Sex | 1.32 | 0.25 |
| Global | 6.05 | 0.11 |
| **NDEV a-syn cutpoint + Age + Sex** |  |  |
| NDEV a-syn cutpoint | 0.94 | 0.33 |
| Age | 3.44 | 0.06 |
| Sex | 1.35 | 0.25 |
| Global | 6.39 | 0.09 |
| **a-syn SAA** | 0.72 | 0.4 |
| **A-syn SAA + Age + Sex** |  |  |
| A-syn SAA | 0.79 | 0.37 |
| Age | 0.87 | 0.35 |
| Sex | 0.72 | 0.40 |
| Global | 2.87 | 0.41 |
| **A-syn SAA and log[miR-7-5p] cut** |  |  |
| Imputation 1 | 0.58 | 0.45 |
| Imputation 2 | 0.79 | 0.37 |
| Imputation 3 | 0.68 | 0.41 |
| Imputation 4 | 0.58 | 0.45 |
| Imputation 5 | 3.04 | 0.081 |
| **A-syn SAA and log[miR-7-5p] cut + Age + Sex** |  |  |
| **Imputation 1** |  |  |
| A-syn SAA and log[miR-7-5p] | 0.91 | 0.34 |
| Age | 0.66 | 0.42 |
| Sex | 0.50 | 0.48 |
| Global | 2.03 | 0.57 |
| **Imputation 2** |  |  |
| A-syn SAA and log[miR-7-5p] | 1.19 | 0.27 |
| Age | 0.63 | 0.43 |
| Sex | 0.44 | 0.51 |
| Global | 2.28 | 0.52 |
| **Imputation 3** |  |  |
| A-syn SAA and log[miR-7-5p] | 1.10 | 0.29 |
| Age | 0.59 | 0.44 |
| Sex | 0.46 | 0.50 |
| Global | 2.17 | 0.54 |
| **Imputation 4** |  |  |
| A-syn SAA and log[miR-7-5p] | 0.91 | 0.34 |
| Age | 0.66 | 0.42 |
| Sex | 0.50 | 0.48 |
| Global | 2.03 | 0.57 |
| **Imputation 5** |  |  |
| A-syn SAA and log[miR-7-5p] | 4.02 | **0.045** |
| Age | 0.68 | 0.41 |
| Sex | 0.48 | 0.49 |
| Global | 5.06 | 0.17 |
| **Age** | 0.24 | 0.63 |
| **Sex** | 1.58 | 0.21 |
